# Polyethylene Glycol Loxenatide Injection (GLP-1) Protects Vascular Endothelial Cell Function in Middle-Aged and Elderly Patients With Type 2 Diabetes by Regulating Gut Microbiota

**DOI:** 10.3389/fmolb.2022.879294

**Published:** 2022-06-15

**Authors:** Fengwu Chen, Lina He, Jilin Li, Shuhui Yang, Bangzhou Zhang, Dan Zhu, Zezhen Wu, Shuo Zhang, Ducheng Hou, Cong Ouyang, Jianfeng Yi, Chuanxing Xiao, Kaijian Hou

**Affiliations:** ^1^ The First Affiliated Hospital of Shantou University Medical College, Shantou, China; ^2^ Department of Endocrine and Metabolic Diseases, Longhu People’s Hospital, Shantou, China; ^3^ Key Laboratory for Research on Active Ingredients in Natural Medicine of Jiangxi Province, Yichun University, Yichun, China; ^4^ Department of Cardiology, The Second Affiliated Hospital of Shantou University Medical College, Shantou, China; ^5^ Department of Endocrine and Metabolic Diseases, Shantou Central Hospital, Shantou, China; ^6^ School of Pharmacy, Fujian University of Traditional Chinese Medicine, Fuzhou, China; ^7^ School of Basic Medical Science, Central South University, Changsha, China; ^8^ Center for Research and Development, Xiamen Treatgut Biotechnology Co., Ltd., Xiamen, China; ^9^ Department of Gastroenterology, The Second Affiliated Hospital of Fujian University of Traditional Chinese Medicine, Fuzhou, China

**Keywords:** GLP-1, type 2 diabetes mellitus, gut microbiota, vascular endothelial cells, middle-aged

## Abstract

**Objective:** To evaluate the protective effect of Polyethylene Glycol Loxenatide Injection (Glucagon-like peptide-1, GLP-1) on endothelial cells from middle-aged and elderly patients with newly diagnosed or poorly controlled type 2 diabetes mellitus (T2DM). GLP-1 weekly formulation was analyzed for cardiovascular disease protection and correlated with intestinal flora.

**Design:** Stool samples were collected from middle-aged and elderly patients with new-onset or poorly controlled type 2 diabetes in Longhu People’s Hospital and Shantou Central Hospital from June 2019 to November 2019. Samples were collected at week 0, 4, and 8 of treatment with GLP-1 weekly formulations. Samples were analyzed for metagenomic sequencing. Analysis was performed to compare the characteristics of the gut microbiota at week 0, 4, and 8 of GLP-1 treatment and to correlate different microbiota with characteristic clinical parameters.

**Results:** Statistical differences were found in blood glucose lowering, cardiovascular endothelial, and inflammation-related indices between week 0 and W4 and in blood glucose lowering and cardiovascular endothelial indices from week 0 to 8 in the newly diagnosed or poorly controlled type 2 diabetic patients treated with GLP-1. Changes in gut microbiota at week 0, 4, and 8 after using GLP-1 were not statistically different, but had an overall trend of rising and then falling, and with different bacteria, that were correlated with different clinical indicators.

**Conclusion:** GLP-1 improves endothelial cell function indicators in middle-aged and elderly diabetic patients, which may be related to its alteration of the population numbers of gut microbiota such as *Acinetobacter*, *Eubacterium ramulus ATCC 29099*, and *Bacteroides_faecis*. This study provides a guidance for the treatment of type 2 diabetic patients.

## 1 Introduction

With the changes in human lifestyle, the prevalence of diabetes is increasing and has become a very serious public health problem worldwide and in China. Although the pathogenesis of diabetes is controversial, it is generally believed to be related to host genes, environment, diet structure, and gut microbiota dysbiosis. In recent years, many studies have revealed that the occurrence, development, and prognosis of diabetes may be associated with gut microbiota (Ma et al., 2019).

The human gastrointestinal tract contains normally a large number of normal microbiota, mainly composed of the Firmicutes, Bacteroidetes, Actinomycetes, Aspergillus, and Wolbachia phyla (Lozupone et al., 2012). The type and quantity of gut microbiota in the guts of diabetic patients are somewhat different compared to normal people (Jayalakshmi et al., 2009; Sedighi et al., 2017). For example, Zhang et al. found a greater number of butyric acid-producing bacteria Akkermansia muciniphila and Faecalibacterium prausnitzii in the normal population than in the pre-diabetic population, the number of anthropoid bacteria was only half of that in the pre-diabetic and the abundance of Wolbachia bacteria was significantly reduced in the diabetic population (Larsen et al., 2010). Moreover, Wu et al. (2010) respectively reported that *Bacillus* or *Bifidobacteria* was significantly decreased in T2DM compared to that of the normal healthy controls. In recent years, it has been found that human gut microbiota is involved in the development of obesity, insulin resistance, and diabetes through different mechanisms, and that many blood glucose-lowering drugs cause changes in gut microbiota. For example, a study (Cao, 2015) found a significant increase in the number and proportion of enterobacteriaceae in type 2 diabetes patients after treatment with acarposose compared with the control group, reaching 71%. Xu et al. conducted high-throughput sequencing analysis on 68 patients with type 2 Diabetes Mellitus (T2DM) (Xu et al., 2013) and found that the number of bifidobacteria in the oral acarbose treatment group increased significantly compared with the control group after 3 months. This was consistent with the results of Su, Aitken and Gerwitz’s study, that is, the number of bifidobacteria in the intestinal microflora of T2DM patients treated with acarbose was significantly higher than that of the control group (Aitken and Gewirtz, 2013; Su et al., 2015). Sun, 2014 found that patients with T2DM treated with metformin showed significant changes in the community composition of their intestinal flora, with an increase in species and no significant difference in the dominant bacteria compared to healthy individuals. Burton et al. (2015) found that the diversity and composition of the gut microbiota changed significantly during metformin treatment, and the use of metformin in combination with gastrointestinal microbial modulators may increase patient tolerance to metformin. Lee and Ko (2014), using mice as experimental subjects, found that the number of *Bacteroidetes* in mice on high-fat diet was significantly reduced compared with the control group while the number of *thick-walled bacteria* was significantly increased compared with the control group, and the number of mimics in mice on high-fat diet was significantly increased after treatment with metformin and was close to that in mice on non-high-fat diet. Hwang et al. (2015) found that the application of liraglutide in diabetic rats led to decrease in the number of microbiota on top of the decrease in the number of high-fat diet microbiota, suggesting a connection between liraglutide and gut microbiota. The above experimental results suggest that some drug treatments for diabetes can cause changes in gut microbiota.

In recent years, a new class of therapeutic drugs targeting the effect of enteroglucagon has been used in clinical practice. The drugs are divided into two types according to mechanism: glucagon-like peptide 1 receptor agonists and dipeptidyl peptidase (DPP-4) inhibitors. GLP-1 agonists have been shown to not only reduce fasting blood glucose (FBG) and 2 h postprandial blood glucose (2hPG), but also reduce body weight, control eating, and protect the function of pancreatic islet B cells (Nadkarni et al., 2014) Representative GLP-1 agonists are lalutide and exenatide. DPP-4 inhibitors can limit the degradation of endogenous GLP-1 (Pratley et al., 2012), and representative drugs include sitagliptin, saxagliptin, liragliptin, and vigliptin. DPP-4 inhibitors are used to enhance glucose-dependent insulin secretion and lower blood glucose by inhibiting the degradation of GLP-1 and increasing fasting and postprandial GLP-1 levels (Nyborg et al., 2012; Xu and Jin, 2014). In terms of plasma half-life, exenatide has a short half-life and is suitable for a twice daily dosing regimen, liraglutide has a long half-life and is suitable for once daily, mainly because the self-linking effect slows down its absorption and binds to albumin and improves its stability to DPP-4 and neutral endopeptidase (NEP). Benalutide has a short half-life and is suitable for a three times daily dosing regimen. The number of doses, gastrointestinal discomfort (including diarrhea, dyspepsia, gastrointestinal reflux disease, nausea, vomiting) is more serious, while multiple injections of dosing, the painful feeling of needle injection, etc. will greatly reduce the patient’s long-term compliance with the drug, thus making the drug less effective (de Moraes and Layton, 2016).

On 3 January 2018, the first glucagon-like peptide-1 receptor agonist weekly formulation in China was officially approved by the State Food and Drug Administration, providing a new therapeutic option for improving glycemic control in patients with type 2 diabetes. The GLP-1 weekly formulation can greatly reduce the frequency of dosing, reduce gastrointestinal adverse effects, and increase the stability of the drug and improve patient compliance, which will provide a new treatment option for most Chinese patients with T2DM (Zhou et al., 2019). Currently, the GLP-1 receptor agonist weekly formulations are available in the United States, Europe, Japan, Korea, Hong Kong, Taiwan, and many other places, and the overall safety is good in the clinics. In addition, studies indicate that GLP-1 analogs, including liraglutide, reduce the risk of cardiovascular events in T2DM due to the expression of GLP-1 receptor) on different cell types, including endothelial cells and immune cells (Helmstädter et al., 2020). Other studies show that the potential cardioprotective effects of GLP-1 can be attributed to their multiple non-glycemic actions in the cardiovascular system, including weight loss, lower blood pressure, improved lipid profile, and direct effects on cardiac and vascular endothelial cells (Andrikou et al., 2019). However, its specific efficacy, safety, and effect on gut microbiota with lowering blood glucose have not been described. There is a lack of data from large-scale prospective studies in our country on the efficacy, safety, compliance, and effects on gut microbiota of GLP-1 weekly preparations in hypoglycemia. Whether the glucose-lowering index of GLP-1 weekly preparation and the protective effect on cardiovascular disease are also related to the change of gut microbiota has not been shown.

In the treatment of type 2 diabetic patients with GLP-1 weekly formulation, we found that the efficacy varied from individual to individual and that patients were accompanied by different degrees of digestive symptoms. Considering the presence of extra-islet glucose control factors, we hypothesized that gut microbiota may influence the therapeutic effect of GLP-1 weekly formulation by affecting the intestinal inflammatory response. To test this hypothesis, this study was proposed to include patients with T2DM who were newly diagnosed or had poor blood glycemic control on metformin alone, who met the enrollment criteria, in an open prospective study. Through an 8-weeks pharmacological intervention, T2DM patients were compared before and after 4, and 8 weeks of treatment for changes in gut microbiota, and related metabolic, vascular endothelial cell function, and inflammatory factors. To support the hypoglycemic effect of GLP-1 weekly preparations and the cardiovascular disease protective effect and the cardiovascular disease protective effect. The results show that GLP-1 improves endothelial cell function indicators in middle-aged and elderly diabetic patients, which may be related to its alteration of the population numbers of specific bacteria in the intestinal microbiome.

## 2 Materials and Methods

### 2.1 Patient Recruitment

#### 2.1.1 Clinical Patient Enrollment

Patients with T2DM who were newly diagnosed clinically or poorly controlled by metformin alone were characterized based on WHO 1999) diagnostic criteria for diabetes mellitus: typical diabetic symptoms + random blood glucose ≥ 11.1 mmol/L; diabetic symptoms + fasting blood glucose (FPG) ≥ 7.0 mmol/L; diabetic symptoms + 2 h postprandial blood glucose ≥ 11.1 mmol/L; oral 75 g anhydrous glucose loading test (OGTT), 2hPG ≥ 11.1 mmol/L confirmed the diagnosis of diabetes mellitus. Diabetic symptoms refer to irritable and excessive drinking, polyuria, polyphagia, and unexplained weight loss. For those without diabetes, only one blood glucose value meets the diagnostic criteria for diabetes, and the diagnosis must be confirmed by rechecking on another day. Random blood glucose refers to any time of the day, regardless of the time of the last meal and food intake; fasting status refers to no calorie intake for at least 8 h.

A total of 12 volunteers were recruited, and the enrolled investigators maintained records of the pre-screened subjects, or a subject screening log. The clinical study followed the Declaration of Helsinki and the official Chinese regulations for clinical research studies. Subjects were enrolled in the clinical study only after they voluntarily sign the informed consent form, and patient privacy was maintained and ensured by the investigator.

#### 2.1.2 Patient Recruitment Standards

Subjects included in this study met the following criteria: male and female subjects were 20 to 75 years old; patients with newly diagnosed T2DM had not been treated with oral hypoglycemic agents or insulin, or patients with T2DM were being treated with metformin but exhibited poor blood glucose control; 6.3% ≤ HbA1c ≤ 10.5%; Fasting C-peptide (FCP) > 1 nmol/L.

Participation and cooperation in the study was voluntary, and all subjects signed an informed consent form.

#### 2.1.3 Exclusion Criteria

Subjects were not included in this study if any of the following exclusion criteria were met: 1) Patients with other types of diabetes mellitus rather than T2DM; 2) Those with severe combined diabetic complications such as diabetic ketoacidosis, hyperosmolar hyperglycemic syndrome, or lactic acidosis; 3) Patients with clinically significant hepatobiliary disease, including but not limited to, chronic active hepatitis and/or severe hepatic insufficiency, cirrhosis, glutamic aminotransferase (ALT) or glutamic oxalacetic aminotransferase (AST) > 3 times the upper limit of normal (150 U/L), or serum total bilirubin (TB) > 34.2 μmol/L (>2 mg/dl); 4) Patients with the following history of renal disease or features associated with renal disease: history of unstable or rapidly progressive renal disease, patients with moderate/severe renal impairment or end-stage renal disease, glomerular filtration rate estimate (eGFR) < 60 ml/min/1.73 m^2^, serum creatinine (Cr) ≥ 133 μmol/L (≥1.50 mg/dl) in male subjects and serum Cr ≥ 124 μmol/L (>1.40 mg/dl) in female subjects; 5) Any of the following cardiovascular conditions: myocardial infarction, cerebral infarction, cardiac surgery or revascularization (coronary artery bypass grafting/percutaneous transluminal coronary angioplasty), unstable angina, congestive heart failure (New York Heart Association class III or IV), transient ischemic attack, or significant cerebrovascular disease within the last 12 weeks; 6) History of gastrointestinal disease or surgery, including intestinal obstruction, intestinal ulcer, bariatric surgery or girdle surgery, gastrointestinal anastomosis, or bowel resection; 7) Pregnant women who were breastfeeding; 8) Urinary tract infection within the last 2 weeks; 9) Subjects who are in the judgment of the investigator unlikely to comply with the protocol, or patients with serious physical or psychological illnesses that could affect the effectiveness or safety of the study.

### 2.2 Interventions

In 12 subjects who voluntarily participated and signed the relevant consent forms, with T2DM newly diagnosed clinically or poorly controlled by metformin alone and who met the inclusion criteria and did not meet the exclusion criteria, relevant specimens were retained before the use of the GLP-1 weekly preparation, and then the GLP-1 weekly preparation (Polyethylene Glycol Loxenatide Injection 0.2 mg each time, subcutaneously every 1 week) was started. The corresponding indices were checked again after 4 and 8 weeks. Changes in glycated hemoglobin and intestinal flora were recorded before and after 4 and 8 weeks of treatment, as well as changes in the following indicators before and after 8 weeks of treatment: fasting glucose, 2-h postprandial glucose, fasting insulin, 2-h postprandial insulin, fasting C-peptide, 2-h postprandial C-peptide, HOMA-IR index, HOMA-HBCI; weight, BMI, waist circumference, waist-to-hip ratio, body fat percentage, basal metabolic rate, etc. Inflammatory factors examined were: CRP (ultrasensitive C-reactive protein), IL-6, IL-8, MCP-1, TNF-α, IL-1β; Changes in vascular endothelial cell function were measured: peripheral blood plasma EMPs levels, markers of endothelial dysfunction associated with inflammation, including soluble intercellular adhesion molecule (sICAM-1), vascular cell adhesion molecule (VCAM-1) and P Selectin, NO, prostacyclin PGI2, and endothelin-1 (ET-1). In addition, markers of endothelial dysfunction related to thrombosis were examined: tissue factor, tissue-type fibrinogen activator, von Willebrand factor, and fibrinogen activator factor inhibitor (PAI-1) change values. The number of hypoglycemia occurrences, combined medications, and adverse events were recorded throughout the study.

### 2.3 Endpoints

#### 2.3.1 Primary Endpoint

Primary endpoints were HbA1c values and changes in gut microbiota of patients before and after 4 and 8 weeks of treatment.

#### 2.3.2 Secondary Endpoint

Secondary endpoints measured were changes in the following indexes after 4 and 8 weeks of GLP-1 weekly formulation intervention.

(1) Glucose metabolism indexes: fasting/2-h postprandial glucose, fasting/2-h postprandial insulin, fasting/2-h postprandial C-peptide, HOMA-IR index, and HOMA-HBCI.
(2) Obesity-related indicators: weight, BMI, waist circumference, hip circumference, waist-to-hip ratio, basal metabolic rate, and total energy.
(3) Inflammatory factors: CRP (hypersensitive C-reactive protein), IL-6, IL-8, MCP-1, TNF-α, and IL-1β.
(4) Vascular endothelial cell function-related indicators: changes in vascular endothelial cell function, including peripheral blood plasma EMPs levels, markers of endothelial dysfunction related to inflammation: soluble intercellular adhesion molecule, vascular cell adhesion molecule and P-selectin, Nitric Oxide (NO), prostacyclin PGI2, endothelin-1; markers of endothelial dysfunction related to thrombosis, including tissue factor, tissue-type fibrinogen activator, von Willebrand factor, fibrinogen activator factor inhibitor.
(5) The ratio of HbA1c ≤ 7.0%.
(6) The ratio of patients with hypoglycemia and gastrointestinal discomfort (blood glucose ≤ 3.9 mmol/L).

#### 2.3.3 Follow-Up Visit Plans

Follow-up visits were performed in the hospital at W0 (week 0), W4 (weeks 4), and W8 (weeks 8), with a 1-month telephone visit at the end of the study.

### 2.4 Analysis of Intestinal Flora

Fecal samples from T2DM patients were collected on the day of the medical examination and stored in microbiota stabilizer EffcGut (Yang et al., 2020) untill DNA extraction. Fecal genomic DNA was extracted using the QIAamp Fast DNA Stool Mini Kit (Qiagen, CA, United States). DNA samples were fragmented to an insert size of 400 bp for library prearation and sequenced by Illumina Nova seq with PE 150 reagents. Raw reads were trimmed to filter the sequencing adapter, low-quality reads, and the human genome (based on reference hg18). Micorbial gene profiles and KEGG orthologous groups (KOs) were generated by aligning the obtained high-quality reads to the reference gene catalogue as previously described (Li et al., 2014). The taxonomic composition at genus, species and strain levels were processed using MetaPhlAn2 (Segata et al., 2012). The differences in the structural composition and functional prediction of bacterial diversity in the W0 and W4and W8 groups using GLP-1 were compared. At the same time, macro-gene sequencing was performed on some samples and sequence splicing function annotation was performed to explore the role of bacteria in the treatment of T2DM from the species level.

### 2.5 Statistical Analysis

Two independent sample T tests were used to compare the mean difference of HbA1c and the secondary end point indexes. For measurement data with non-normal distribution and microbial eatues, Wilcoxon rank sum test was used for comparison between groups. Spearman correlation was used to the calculate the correlation between clinical data and microbial taxa. Data were visualized using R language (Mair et al., 2015), mainly with packages of reshape2 (Zhang, 2016), ggplot2^1^, ggsignif, ape (Paradis et al., 2004), gridExtra^2^.

## 3 Results

### 3.1 Improvement of Clinical Indicators

In this study, a total of 36 stool samples and clinical data from 12 patients with T2DM were analyzed at W0, 4, and 8 during treatment with GLP-1. The patients had a mean age of 62 years at baseline and a mean BMI of 21.57. This study identified clinical indicators that showed significant changes between W0 and W4, and between W0 and W8. The glucose lowering indicators with significant differences in improvement at W4 compared to W0 were 2HPG, FCP; the cardiovascular endothelium-related indicators that improved were tissue factor, PAI-1, Endothelin-1, von Willebrand factor, tissue type fibrinogen activator, Prostacyclin PGI2, and LDLC. One weight-related indicator improved, BMI (kg/m2), and one inflammation-related indicator improved, IL-6 (Table 1). The glucose lowering indices with significant difference improvement at W8 compared with W0 were: HbA1c, islet β-cell function index [HOMA-β: 20 * FINS/(FPG-3.5)], FBG, 2HPG, and FINS. Cardiovascular endothelium related indexes improved were: tissue factor, albumin, von Willebrand factor, tissue type fibrinogen activator, and adenosine dehydrogenase (Table 2). These clinical indicators, which were statistically different between 0 to W4, and between 0 and W8, are markers of endothelial dysfunction associated with diabetes and thrombosis, indicating that GLP-1 has a good hypoglycemic effect on type 2 diabetic patients and a protective effect on vascular endothelial function.

**TABLE 1:** Clinical information for patients treated with GLP-1 from W0 toW4.

Participants, *n* = 12	W0–W4 (T test)			
Clinical information	W0_sd	W4_sd	*p*-value_04t	p.adjust_04t
PAI-1	61.23 ± 6.4	57.65 ± 6.52	5.40E-06	0.00019718
Tissue factor	56.69 ± 6.46	52.78 ± 5.99	3.60E-05	0.00065758
Endothelin-1 (ET-1)	96.5 ± 20.28	91.85 ± 19.82	0.000139623	0.00203849
Von willebrand factor	192.54 ± 16.03	182.98 ± 14.9	2.61E-06	0.00019078
Tissue type fibrinogen activator	16.68 ± 2	17.86 ± 1.88	2.33E-05	0.00056622
Prostacyclin PGI2	99.55 ± 19.09	106.7 ± 22.97	0.001445291	0.01758438
LDLC	3.17 ± 1.05	2.76 ± 0.87	0.002960829	0.02879347
Albumin	43.03 ± 3.49	44.21 ± 3.78	0.31165418	0.517062617
Apolipoprotein A (g/L)	1.39 ± 0.3	1.38 ± 0.24	0.896860597	0.909316994
Glucose	10.83 ± 4.32	8.83 ± 2.29	0.07037148	0.210221666
Age	62.01 ± 10.61	62.01 ± 10.61	NA	NA

Note: The data were expressed as mean ± SD.

**TABLE 2:** Clinical information for patients treated with GLP-1 from W0 to W8.

Participants, *n* = 12	W0–W4 (T test)			
Clinical information	W0_sd	W8_sd	pvalue_08t	p.adjust_08t
PAI-1	61.23 ± 6.4	54.55 ± 6.15	1.64E-08	5.98E-07
Tissue factor	56.69 ± 6.46	49.45 ± 5.22	1.23E-07	2.99E-06
Endothelin-1 (ET-1)	96.5 ± 20.28	84.85 ± 17.72	3.77E-07	6.88E-06
Von willebrand factor	192.54 ± 16.03	175.77 ± 14.51	1.99E-09	1.45E-07
Tissue type fibrinogen activator	16.68 ± 2	18.89 ± 2.42	2.24E-05	0.000327513
Prostacyclin PGI2	99.55 ± 19.09	118.68 ± 31.65	0.00071005	0.007404807
LDLC	3.17 ± 1.05	2.32 ± 0.82	0.001058853	0.009662035
Albumin	43.03 ± 3.49	47 ± 2.89	0.000343558	0.004179959
Apolipoprotein A (g/L)	1.39 ± 0.3	1.47 ± 0.25	0.492811875	0.609750286
Age	62.01 ± 10.61	62.01 ± 10.61	NA	NA

Note: The data were expressed as mean ± SD.

### 3.2 Changes in Gut Microbiota in Middle-Aged and Elderly Patients With T2DM Treated With GLP-1 at 0, 4, and W8

**FIGURE 1:**
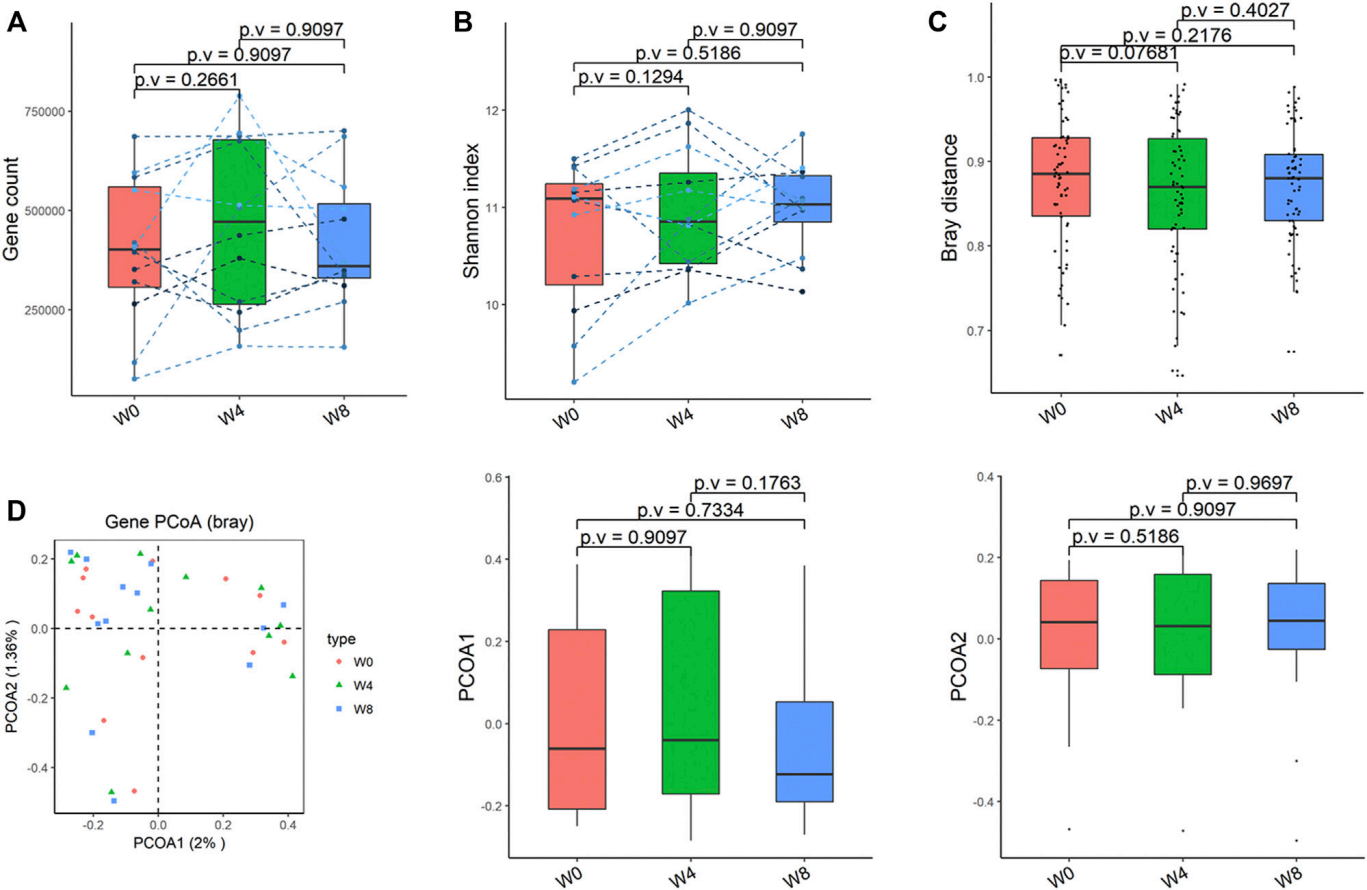
Alpha-diversity, beta-diversity, and principal component analysis at the gene level. **(A)** Gene count detected was not significantly different in comparisons between W0 and W4, W0 and W8, and W4 and W8. **(B)** Shannon index was not significantly different in comparisons between W0 and W4, W0 and W8, and W4 and W8 **(C)** No significant difference in beta diversity based on Bray-Curtis (Bray) distance for W0 vs W4, W0 vs W8, and W4 vs W8. **(D)** PCoA analysis based on Bray distance showed that the first principal component was not significantly different in W0 vs W4, W0 vs W8, and W4 vs W8, nor the second component.

**FIGURE 2:**
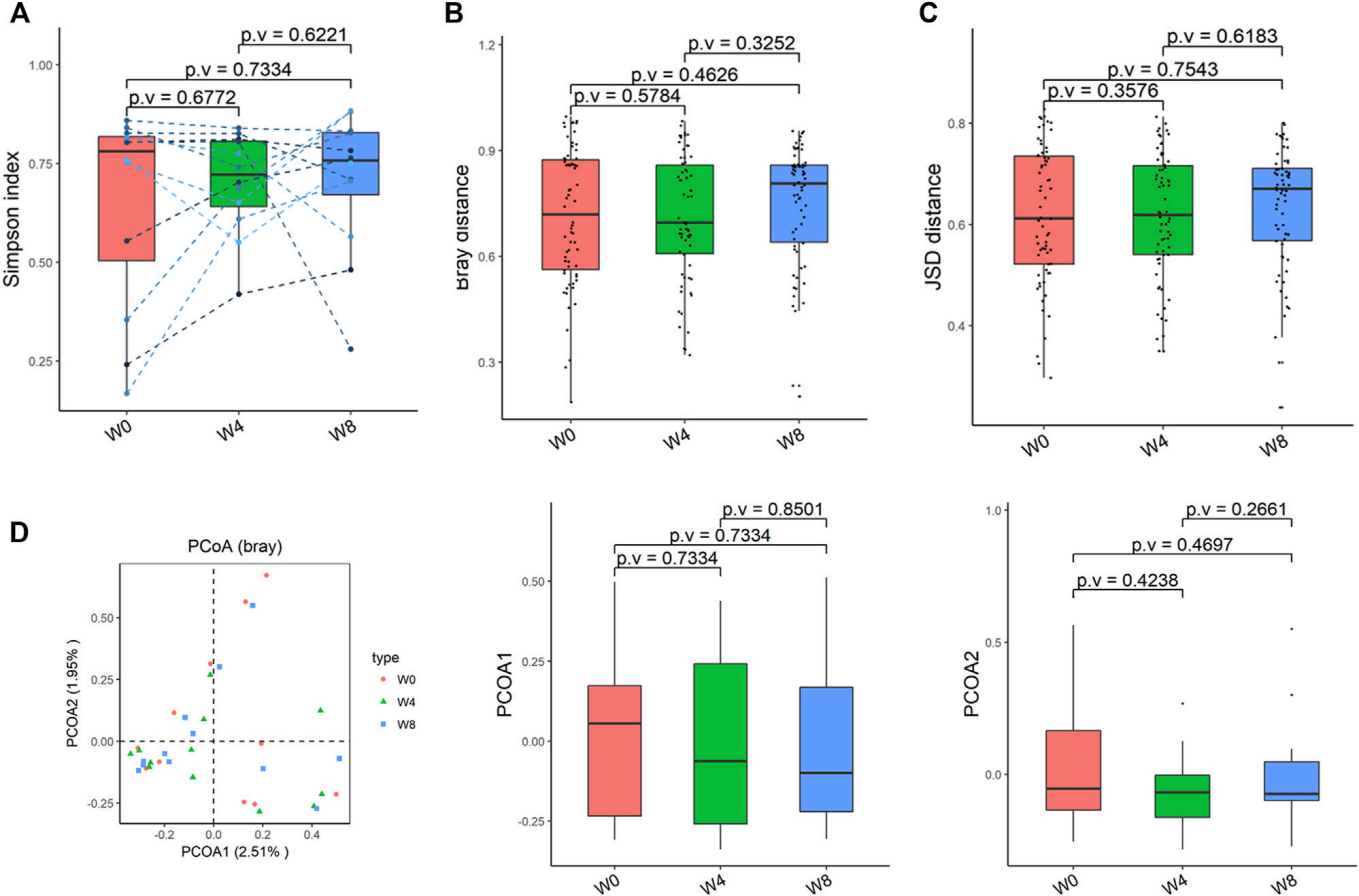
Alpha-diversity, beta-diversity, and principal component analysis at the species level. **(A)** Simpson index was not significantly different in comparisons between W0 and W4, W0 and W8, and W4 and W8. **(B)** No significant difference in beta diversity based on Bray-Curtis (Bray) distance for W0 vs W4, W0 vs W8, and W4 vs W8. **(C)** The Jensen-Shannon Divergence (JSD) distance-based beta diversity was not significantly different in W0 vs W4, W0 vs W8, and W4 vs W8. **(D)** PCoA analysis based on Bray distance showed that the first principal component was not significantly different in W0 vs W4, W0 vs W8, and W4 vs W8, nor the second component.

**FIGURE 3:**
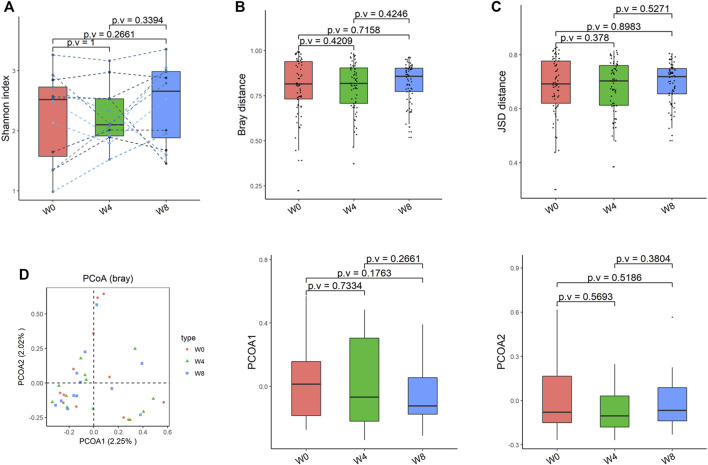
Alpha-diversity, beta-diversity, and principal component analysis at the species level. **(A)** Shannon index was not significantly different in comparisons between W0 and W4, W0 and W8, and W4 and W8. **(B)** No significant difference in beta diversity based on Bray-Curtis (Bray) distance for W0 vs W4, W0 vs W8, and W4 vs W8. **(C)** The Jensen-Shannon Divergence (JSD) distance-based beta diversity was not significantly different in W0 vs W4, W0 vs W8, and W4 vs W8. **(D)** PCoA analysis based on Bray distance showed that the first principal component was not significantly different in W0 vs W4, W0 vs W8, and W4 vs W8, nor the second component.

**FIGURE 4:**
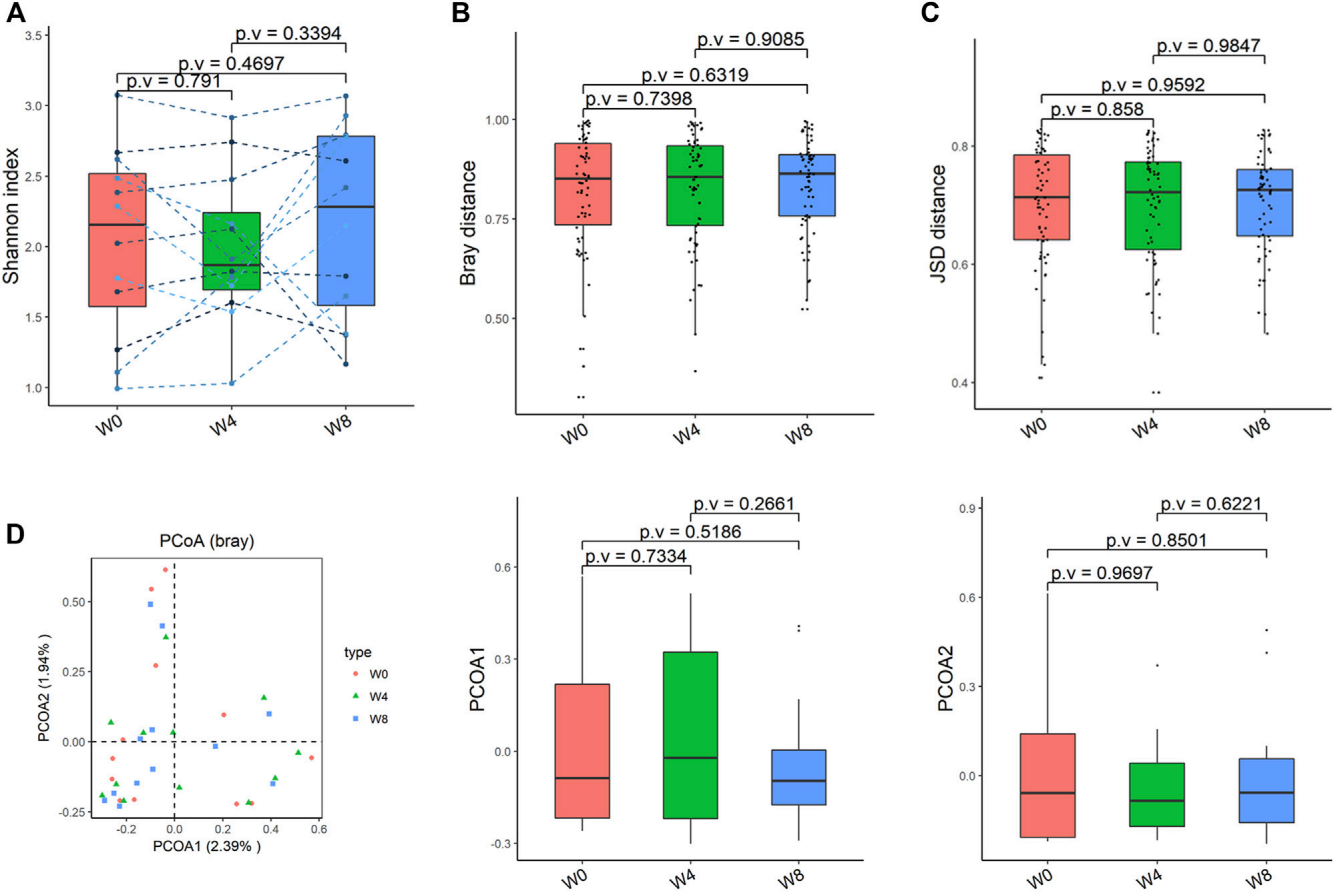
Alpha-diversity, beta-diversity, and principal component analysis at the strain level. **(A)** Shannon index was not significantly different in comparisons between W0 and W4, W0 and W8, and W4 and W8. **(B)** No significant difference in beta diversity based on Bray-Curtis (Bray) distance for W0 vs W4, W0 vs W8, and W4 vs W8. **(C)** The Jensen-Shannon Divergence (JSD) distance-based beta diversity was not significantly different in W0 vs W4, W0 vs W8, and W4 vs W8. **(D)** PCoA analysis based on Bray distance showed that the first principal component was not significantly different in W0 vs W4, W0 vs W8, and W4 vs W8, nor the second component.

**FIGURE 5:**
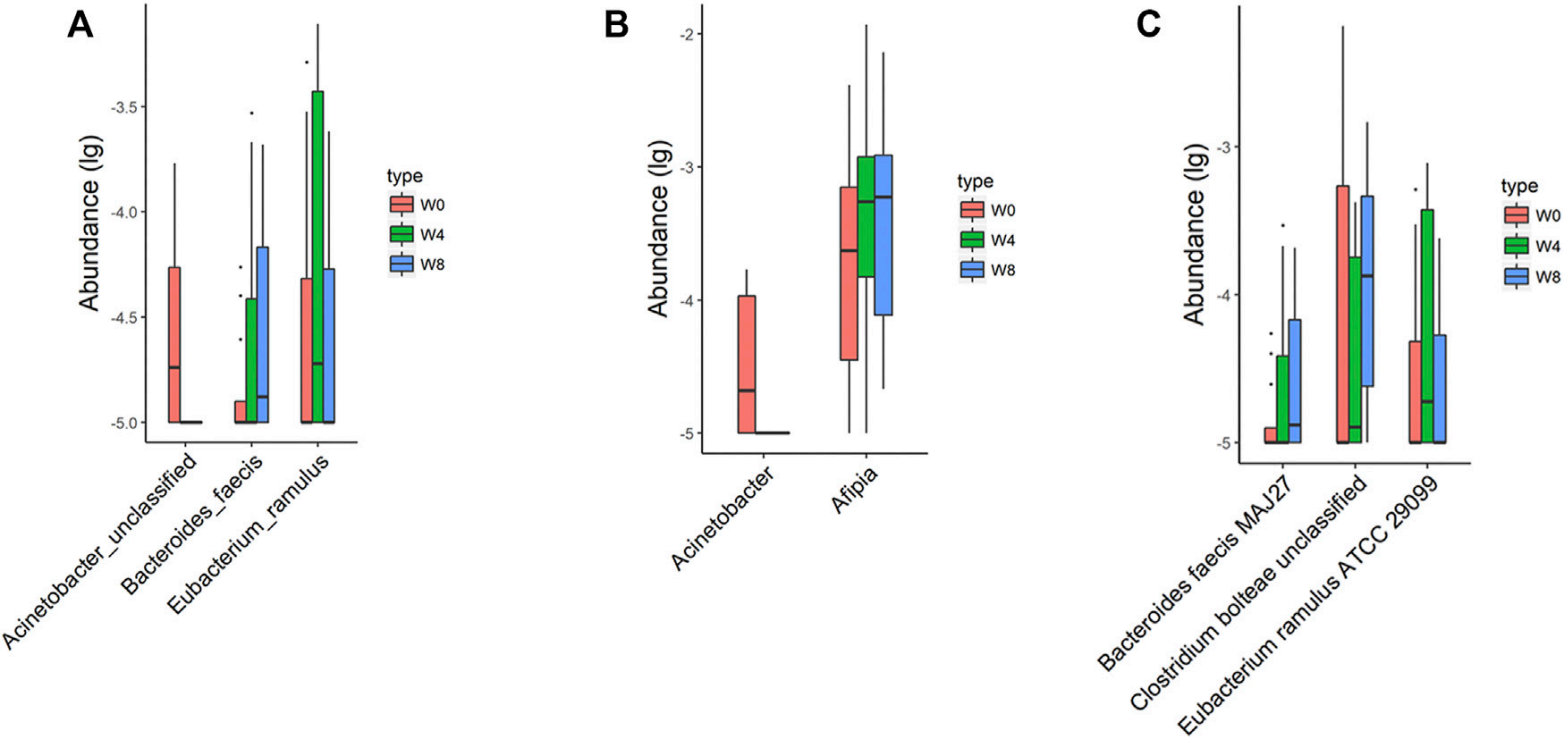
Significantly different taxa at levels of species **(A)**, genus **(B)**, and strain **(C)** in comparisons between W0 vs W4, W0 vs W8, and W4 vs W8.

Comparing the differences in the intestinal flora diversity and function prediction of T2DM patients after GlP-1 treatment at 0, 4, and W8. At the same time, macrogene sequencing was performed on some samples and sequence-splicing function annotation was performed to explore the role of bacteria in the treatment of T2DM at the species level. Diversity analysis at the gene level, T2DM including gene stripes (A), Alpha diversity (B), Beta diversity (C), and principal component analysis (D), was performed as shown in Figure 1. The number of genes showed an increasing trend after W4 of GLP-1 use, but then showed a decreasing trend at W8. There was a trend of change in the number of genes in patients after GLP-1 use. Although this trend was not statistically significant (*p* > 0.05). There was also an upward and then downward trend at the genus (Figure 2) and species and subspecies levels (as shown in Figures 3, 4). In terms of microbial diversity, the overall trend was rising and then declining (see Figure 2). At the genus level, one significant difference can be found for the genera atW0,W4, and W8: *Acinetobacter* (*p* < 0.005); at the species level, one significant difference can be found at W0, W 4, and W8: *Acinetobacter-unclassified* (*p* < 0.005). Two bacteria were significantly different at 0 and W8: *Acinetobacter-unclassified* (*p* < 0.05) and *Bacteroides_faecis* (*p* < 0.05); one bacteria differed significantly at W4 and W8: *Eubacterium_ramulus*. Significant difference analyses at the strain level, in W0 versus W4, W0 versus W8, and W4 versus W8, revealed the following: at W0 and W8 there was a significant difference in the strain level, *Bacteroides faecis MAJ27*; at W4 and W8 there were a significant differences in the strain level of *Clostridium bolteae* (*p* < 0.05) and *Eubacterium ramulus ATCC 29099* (*p* < 0.05) (Figure 5).

**FIGURE 6:**
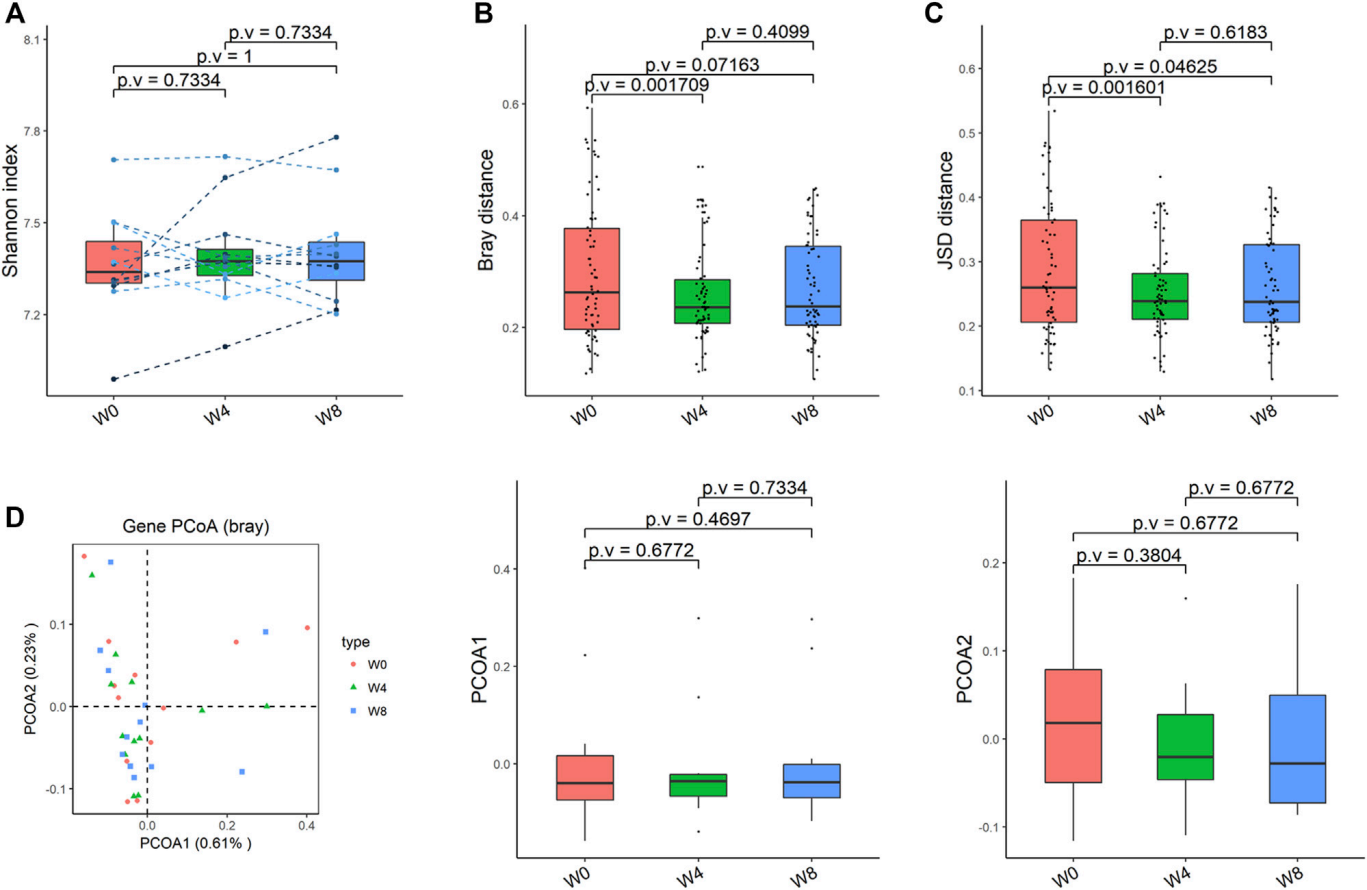
Alpha-diversity, beta-diversity, and principal component analysis at the KO level. **(A)** Shannon index was not significantly different in comparisons between W0 and W4, W0 and W8, and W4 and W8. **(B)** Significant difference in beta diversity based on Bray-Curtis (Bray) distance for W0 vs W4, but not in W0 vs W8, norW4 vs W8. **(C)** The Jensen-Shannon Divergence (JSD) distance-based beta diversity was significantly different in W0 vs W4 and W0 vs W8, but not W4 vs W8. **(D)** PCoA analysis based on Bray distance showed that the first principal component was not significantly different in W0 vs W4, W0 vs W8, and W4 vs W8, nor the second component.

To verify the change of functional pathways, KO—Alpha Diversity, KO—beta Diversity, KO—Principal Coordinate Analysis PCoA (Bray) were analyzed and found that there was changes in the functions with signals, modules, pathways. The beta diversity based on Jensen-Shannon Divergence (JSD) distance was significantly different at 0 and W4 (*p* = 0.001), and 0 and W8 (*p* = 0.046) (Figure 6).

**FIGURE 7:**
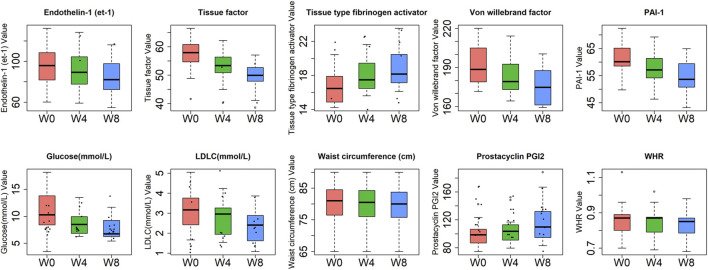
Trends over time in clinical indicators with significant effects on gut microbes.

**FIGURE 8:**
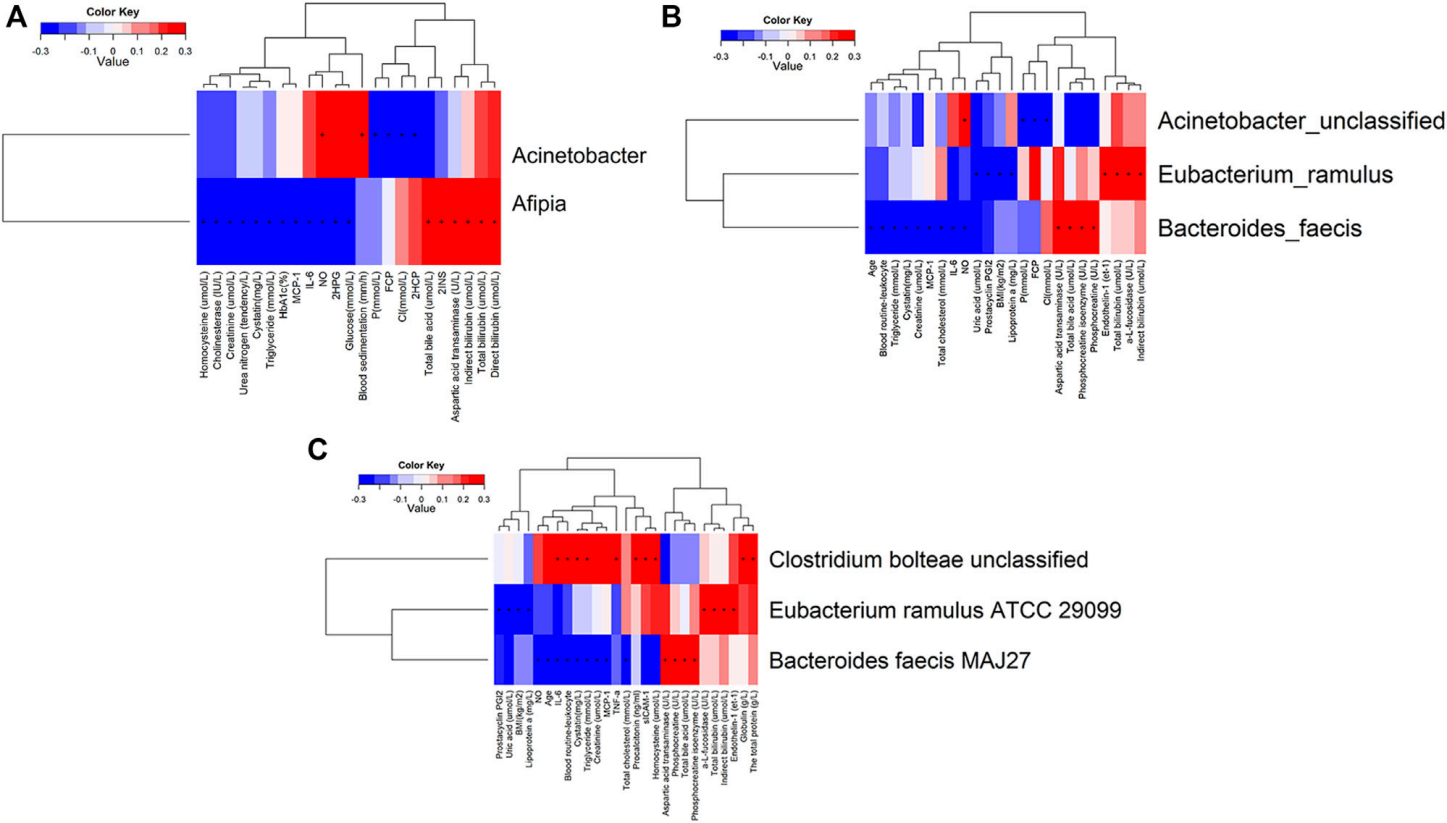
Correlation analysis of significantly different clinical indicators and differential bacteria at levels of genus **(A)**, species **(B)** and strain **(C)** in type 2 diabetic patients.

In addition, we also analyzed the significant differences of phenotypes in W0 and W4, W0 and W8, and W4 and W8, and found that there were 27 significantly different phenotypes in W0 and 4, 23 significantly different phenotypes in W0 and W8, and 18 significantly different phenotypes in W4 and 8. Permanova analysis of significantly different phenotypes based on the bray distance at the gene level showed that 19 phenotypes had a significant effect on gut microbes (*p* < 0.05) as shown in Figures 7, 8. As clearly shown in the Table 3 below, glucose lowering indices and cardiovascular disease related indices correlate with gut microbes.

**TABLE 3:** Clinical indicators associated with intestinal microbiota.

Phenotype	SampleNum	Df	SumsOfSqs	MeanSqs	F. Model	R2	Pr (>F)
Alkaline phosphatase (U/L)	36	1	0.732253123	0.732253123	2.03707352	0.056527163	0.0046
FBG	36	1	0.706773832	0.706773832	1.962101546	0.054560258	0.0059
HbA1c (%)	36	1	0.672513636	0.672513636	1.861782402	0.051915501	0.0098
NO	36	1	0.667862061	0.667862061	1.84820501	0.051556417	0.0099
Glucose (mmol/L)	36	1	0.648365136	0.648365136	1.791407492	0.050051328	0.0132
LDLC (mmol/L)	36	1	0.647663586	0.647663586	1.789367126	0.049997171	0.0138
FCP	36	1	0.637714707	0.637714707	1.760457113	0.049229156	0.0155
Prostacyclin PGI2	36	1	0.641743358	0.641743358	1.772158163	0.049540152	0.0172
PAI-1	36	1	0.626620928	0.626620928	1.728275213	0.048372758	0.0187
Cl (mmol/L)	36	1	0.622514811	0.622514811	1.716378478	0.048055781	0.019
Von willebrand factor	36	1	0.616806148	0.616806148	1.699851811	0.047615094	0.0203
Tissue type fibrinogen activator	36	1	0.593225856	0.593225856	1.631748259	0.045794785	0.0282
MCP-1	36	1	0.566648666	0.566648666	1.555300003	0.043743127	0.0309
Transglutaminase (U/L)	36	1	0.543817812	0.543817812	1.489889395	0.041980672	0.0353
BMI (kg/m^2^)	36	1	0.574320548	0.574320548	1.577334174	0.044335367	0.0388
Tissue factor	36	1	0.567614984	0.567614984	1.558073833	0.043817723	0.0427
2INS	36	1	0.559606295	0.559606295	1.535097846	0.043199483	0.0451
Adenosine dehydrogenase (U/L)	36	1	0.56118808	0.56118808	1.539633447	0.043321591	0.0453
Homocysteine (μmol/L)	36	1	0.529575982	0.529575982	1.44920817	0.040881256	0.0512
Waist circumference (cm)	36	1	0.544807198	0.544807198	1.492719006	0.042057049	0.0583
TNF-a	36	1	0.321946367	0.321946367	0.86653943	0.024853038	0.744
IL-6	36	1	0.272752462	0.272752462	0.731282972	0.021055455	0.8728

With the change of time, there was a decreasing trend of body mass index and vascular damage related indexes at different time points of W0, W4, and W8 of treatment with GLP-1 in type 2 diabetes. This indicates that GLP-1 has a good effect on weight reduction and protection of vascular endothelial cells. The indicators associated with beneficial effects on cardiovascular disease showed an increasing trend, and those associated with harmful effects on cardiovascular disease showed a decreasing trend. The specific indicators of change include Prostacyclin PGI2, tissue-type fibrinogen activator with an increasing trend, while some indicators such as fibrinogen activator inhibitor-1 (PAI-1), endothelin-1 (et -1), and Tissue factor (et al.) showed a decreasing trend. Waist circumference and waist-to-hip ratio showed a decreasing trend. Cycloprost PGI2, endothelin, tissue factor, tissue-type fibrinogen activator, and vascular pseudohemophilic factor are all indicators related to vascular endothelial function, and the changes in these indicators are the main clinical results of the protective effect of GLP-1 on vascular endothelium in middle-aged and elderly diabetic patients, indicating that GLP-1 has a protective effect on vascular endothelium (Figure 8).

### 3.3 Analysis of the Correlation Between Clinical Indicators and Gut Microbiota

Correlation analysis was undertaken to examine clinical indicators with significant differences in T2DM with characteristic differences in the gut microbiota genera, where “+” indicates *p*-value <0.05, and “*” indicates *p*-value <0.001. Clinical indicators with positive correlation and significant differences in the genus *Fusobacterium* were nitric oxide and blood sedimentation. Clinical indicators that were negatively correlated and significantly different from Aphanizomenon spp. were fasting C-peptide and 2-h postprandial C-peptide. Clinical indicators that showed a positive correlation with *Aphanizomenon*. with significant differences were total bile acids, 2-h postprandial C-peptide, aspartate aminotransferase, indirect bilirubin, total bilirubin, and direct bilirubin. Clinical indicators that showed a negative correlation with *Aphanizomenon*. with significant differences were homocysteine, cholinesterase, creatinine, urea nitrogen, cystatin, triglycerides, glycated hemoglobin, monocyte chemokine-1, interleukin-6, nitric oxide, and 2-h postprandial glucose (Figure 8A). Clinical indicators that showed a positive correlation with *Fusobacterium* with significant differences were nitric oxide. Significantly different clinical indicators that negatively correlated with *Fusobacterium* were fasting C-peptide and chloride. Clinical indicators that showed a positive correlation with *Fusobacterium* with significant differences were endothelin 1, a-L-amylase, total bilirubin, and indirect bilirubin. Clinical indicators that showed a negative association with *S. cerevisiae* with significant differences were uric acid, prostacyclin I2, body mass index, and apolipoprotein A. Clinical indicators that showed a positive association with *S. cerevisiae* with significant differences were aspartate aminotransferase, total bile acids, phosphocreatine isoenzymes, and phosphocreatine. Clinical indicators that showed negative correlation with *Acinetobacter* with significant differences were age, leukocytes, triglycerides, cysteine, creatinine, monocyte chemokine-1, total cholesterol, interleukin-6, nitric oxide, and uric acid (Figure 8B). The clinical indicators that showed positive correlation with *Acinetobacter* with significant differences were interleukin-6, leukocytes, triglycerides, globulin, total protein, and cysteine. Clinical indicators that showed positive correlation with *Acinetobacter* with significant differences were a-L-fucosidase, total bilirubin and indirect bilirubin (Figure 8C).

These results indicate that the gut microbiota and indicators related to glucose metabolism and vascular endothelial cell function were improved in T2DM patients after the use of GLP-1, suggesting that GLP-1 has a good hypoglycemic effect and protective effect on vascular endothelial function in patients with T2DM. In terms of microbial diversity, there was no significant difference among groups at 0, 4, and W8, but the overall trend was first increased and then decreased. There are individual species that are significant at the species level. The correlation analysis of clinical indicators and characteristic bacteria showed that the improvement of clinical indicators was closely related to the intestinal microflora (*p* < 0.05), suggesting that the hypoglycemic and endothelial protective effects of GLP-1 are closely related to the gut microbiota. That is, GLP-1 may protect vascular endothelial cell function in middle-aged and elderly patients with T2DM by regulating the gut microbiota.

## 4 Discussion

Many studies shown that patients with T2DM led to impairment in endothelial-dependent vasodilatation, decreased expression of NO, showing a dysfunction of cardiovascular and endothelial. (Néri et al., 2021; Tay et al., 2021; Wang et al., 2021).

In our previously published article (Que et al., 2021), we found a consistent trend toward increased relative abundances of the phyla Firmicutes (class Negativicutes or family Veillonellaceae) and Actinobacteria and decreased relative abundances of Bacteroidetes (class Bacteroidia or family Bacteroidaceae) for T2DM. The relationship between T2DM-associated (enriched or depleted) genera and probiotics shows that *Clostridium* sensu stricto 1 and Blautia were positively correlated with B. breve, and that *Lactobacillus* enriched in T2DM patients was correlated negatively with B. bifidum.

The main change seen in various research is an increase in the amount of opportunistic pathogens (Qin et al., 2012; Karlsson et al., 2013; Allin et al., 2015) such as Akkermansia muciniphila (Qin et al., 2012) and four *Lactobacillus* species (Karlsson et al., 2013). And the decreased levels of the phylum Firmicutes, class Clostridia, Faecalibacterium, Roseburia, butyrate producers (Qin et al., 2012; Karlsson et al., 2013), Akkermansia muciniphila, Roseburia (Salamon et al., 2018) and species of the genus *Clostridium* and Akkermansia muciniphila (Allin et al., 2018).

The results of various studies differ from one another, but, in general, the genera negatively associated with T2D are *Bacteroides*, Bifidobacterium, Faecalibacterium, Akkermansia and Roseburia, and the genera Fusobacteria, Ruminococcus and Blautia are positively connected with this disease (Gurung et al., 2020; Bielka et al., 2022).

In this study, we found that the middle-aged and elderly diabetic patient population showed significant improvement in blood glucose lowering indices and inflammation-related indices after 8 and W4 of GLP-1 use compared to W0. Also, there were significant changes in the indicators related to vascular endothelial cell function, and this improvement was associated with the change in the populations of gut microbiota such as *Acinetobacter*, *afebola*, *Eubacterium twigs*, *Bacteroides*, and *Acinetobacter baumannii*. Furthermore, it was revealed that GLP-1 is not only a hypoglycemic drug, but also has a protective effect on the function of vascular endothelial cells in the middle-aged and elderly diabetic patients (Wang et al., 2013).

In our study, indicators related to vascular endothelial cell function, including Prostacyclin PGI2, tissue-type fibrinogen activator, fibrinogen activator inhibitor-1, endothelin-1, and tissue factor were improved, suggesting that GLP-1 can protect the cardiovascular system in treatment, which is consistent with the results of RCTs with large clinical data and long follow-up (Rutten et al., 2016; Ipp et al., 2017). In Leader’s study, researchers found whether the GLP-1 receptor agonist liraglupeptide reduced the risk of cardiovascular death in a large clinical study of 374 elderly people at high cardiovascular risk. In T2 DM patients with a history of cardiovascular disease or cardiovascular risk factors, GLP-1 treatment significantly reduced the risk of the primary endpoint of cardiovascular death, non-fatal myocardial infarction, and stroke in diabetic patients (Ipp et al., 2017). In contrast, in the SUSTAIN-6 study, a placebo-controlled design was used to enroll a total of 3,297 patients ≥50 years of age with T2DM with the primary endpoint of first major adverse cardiovascular event (including cardiovascular death, nonfatal myocardial infarction, or nonfatal stroke). Results at W4 of follow-up showed a 26% reduction in the risk of MACE with somalutide, again showing that GLP-1 receptor agonists have cardioprotective effects (Ipp et al., 2017). It has also been shown in preclinical studies that both GLP-1 receptor agonists and DPP-4 inhibitors exhibit cardioprotective effects in animal models of myocardial ischemia and ventricular insufficiency through incompletely characterized mechanisms. At the species level, fasting blood glucose is negatively correlated with *Eubacterium ramulus*, *Roseburia inulinivorans*, *Roseburia hominis*, *Eubacterium eligens*, and *Ruminococcus callidus*. This indicates that patients with T2DM and diabetic cardiovascular complications have significant abnormal glucolipid metabolism and gut microbiota dysbiosis, and gut microbiota disorders may play an important role in the pathogenesis and progression of diabetes. In our study, the number of fine fungal bacteria was also changed in T2DM patients with GLP-1, and it was negatively correlated with prostacyclin I2. Prostacyclin I2 inhibits platelet-mediated agglutination process and has a strong vasodilatory effect, so that the production of prostacyclin I2 in the damaged vascular endothelium is reduced during coagulation, which facilitates platelet aggregation. Therefore, the vasoprotective effect of GLP-1 may be produced by regulating the number of fine branching fungi, thus affecting the production of prostacyclin I2.

In the present study we found that the gut microbiota of T2DM patients was significantly altered with the use of GLP-1, and the altered bacteria were: *Acinetobacter*, *afebola*, *Eubacterium twigs*, *Bacteroides*, *Acinetobacter baumannii*. Among them, the changes of *Acinetobacter*, *afebo*, and *Bacteroides* were correlated with NO. In this study after the use of GLP-1, NO of the patients was positively correlated with *Acinetobacter*, and negatively correlated with *afebo* and *Bacteroides*. NO is the primary endothelial regulator for local vascular tone and inhibits the production and action of other vasoactive factors, such as the vasoconstrictors prostaglandin and ET-1. In the early stages of vascular disease, when NO-mediated responses are impaired, prostacyclin and endothelium-dependent hyperpolarization are helpful in compensating for the damage. However, as the ability of endothelial cells to release NO gradually decreases, the production of endothelium-derived cyclic oxygenase-dependent contractile factor, ET-1, increases and helps vasoconstriction. At the same time, the protein expression of endothelial cell adhesion molecules (e.g., vascular cell adhesion molecule 1, intercellular adhesion molecule 1, E-selectin) is enhanced due to the diminished protective effect of NO, which promotes leukocyte adhesion and infiltration, resulting in increased E-selectin on endothelial cells and promoting leukocyte adhesion and infiltration (Vanhoutte, 2009; Vanhoutte et al., 2016; Vanhoutte et al., 2017). The elevated NO facilitates repair of endothelial injury. InT2DM patients in this study, NO levels were elevated after the administration of GLP-1, and the levels of NO were positively correlated with *Acinetobacter*, and negatively correlated with *afebo* and *Bacteroides*, which means the levels of *Acinetobacter* decreased while the levels of *afebo* and *Bacteroides* increased (Madsen et al., 2019). In a Japanese study of gut microbiota in obese and non-obese subjects, T-RFLP analysis showed that obese subjects had a significantly lower number of phylum *Bacteroides* and a significantly lower ratio of thick-walled phylum to phylum *Bacteroides* compared to non-obese subjects. Bacterial diversity was significantly higher in obese subjects compared to non-obese subjects (Kasai et al., 2015). In contrast, in animal experiments, we also observed an increase in SCFA-producing bacteria in experimental rats after GLP-1 (liraglutide) injection, including the genera *Anaplasma*, *Trichophyton*, and *Bifidobacterium* (Zhang et al., 2018). GLP-1 (liraglutide) significantly altered the overall composition of the intestinal microbiota, consistent with its weight loss effect (Wang et al., 2016). These reports are all consistent with our findings. It has been shown that a decrease in NO can lead to endothelium-dependent reduced vasodilatory function and affect the metabolism and function of the vascular wall, while overproduction of reactive oxygen species is one of the important factors contributing to endothelial dysfunction (Smits et al., 2021). Gut microbiota can promote atherosclerosis and vascular endothelial cell dysfunction. Vascular endothelial cell dysfunction, marked by impaired endothelium-dependent vasodilation, is an early indicator of atherosclerosis, and the expression of vascular microRNA-204 (miR-204) is remotely regulated by the flora, with miR-204 targeting downregulation of deacetylase 1, resulting in impaired endothelial cell function (Vikram et al., 2016).

In our study, type 2 diabetic patients with GLP-1 showed a decrease in BMI, an increase in *Bacteroides* levels, and a negative correlation with NO levels, suggesting that the use of GLP-1 in type 2 diabetic patients may alter the gut microbiota of patients, and that GLP-1 may exert vascular endothelial protective effects by increasing the levels of fecal *Bacteroides* in type 2 diabetic patients, regulating their body weight, causing a decrease in their BMI levels, and downregulating their NO levels, thereby inhibiting the production and effects on other vasoactive factors.

In terms of microbial alpha and beta diversity, alpha diversity is an ecological indicator of the abundance and diversity of microbiota in a sample (for example, the abundance distribution of detected microorganisms in each sample); beta diversity indicates differences in composition between samples (for example, the composition of microbiota in different subjects from different treatment groups). It was found that there was an overall trend of increasing and then decreasing microbial diversity. This indicates a gradual process of increasing microbiota diversity over the course of 0-W4 with GLP1. Previous animal studies have also (Li et al., 2014) shown that probiotics can significantly improve patients’ blood glucose and lipid indexes, and improve morphological changes in the pancreas, liver, and kidneys. In this study, differential analysis of gut microbiota at W0, W4, and W8 with GLP-1 weekly preparations revealed significantly different genera: *Acinetobacter* (*p* = 0.000982716). In the present study, there was a decreasing trend in the abundance of *Acinetobacter* at 0, 4, and W8, and a decreasing trend in the indicators related to cardiovascular adverse effects and an increasing trend in the indicators related to beneficial effects, including an increasing trend in prostacyclin PGI2, tissue-type fibrinogen activator, and a decreasing trend in some indicators such as fibrinogen activator inhibitor-1 (PAI-1), ET-1, and tissue factor. PAI-1, e ET-1, and tissue factor showed a decreasing trend. This is consistent with the trend of the indicators harmful to the endothelium and opposite to the trend of the indicators beneficial to the endothelium. This leads us to speculate that the reduction of *Acinetobacter baumannii* may be an indicator of improved cardiovascular risk in elderly T2DM patients, and that the improvement of GLP-1 as an indicator of vascular endothelial function in middle-aged and elderly T2DM patients may depend on the reduction of *Acinetobacter baumannii* in the intestinal tract of patients.

To conclude, the present study shows that the hypoglycemic effect of GLP-1 as a drug in diabetic patients and the protective effect of cardiovascular disease are worthy of recognition. Most notably, we found that certain characteristic bacteria are correlated with relevant clinical indicators, such as *Acinetobacter*, *Afebola*, *Eubacterium twigs*, *Bacteroides*, *Acinetobacter baumannii*. The level of NO of the patients was positively correlated with the genus *Acinetobacter* and negatively correlated with the genus *Afebola* and *Bacteroides*. The improvement of vascular endothelial cell function index of middle-aged and old-aged diabetic patients by GLP-1 may be related to its change of the number of gut microbiota of the patients. Therefore, we suggest that the cardioprotective function of GLP-1 in diabetic patients may be correlated with the specific differential bacteria of the gut microbiota.

## Data Availability

The raw sequencing data supporting the conclusions of this article were deposited in the NCBI Sequence Read Archive (SRA) database under bioproject number PRJNA809514. (https://www.ncbi.nlm.nih.gov/sra/PRJNA809514).

## Footnotes

1 <*ggplot2- Elegant Graphics for Data Analysis.pdf*>.

2 <*gridExtra.pdf>*.
