# Supplementary material for: Polyethylene Glycol Loxenatide Injection (GLP-1) Protects Vascular Endothelial Cell Function in Middle-Aged and Elderly Patients With Type 2 Diabetes by Regulating Gut Microbiota
Source: Front Mol Biosci. 2022 Jun 15;9:879294. doi: 10.3389/fmolb.2022.879294 (PMC9240776; doi:10.3389/fmolb.2022.879294)
Supplement: Supplementary file 7 [file DataSheet1.DOCX]

Thank you very much for your information. The raw sequencing data of paper ID 879294 have been deposited in the NCBI Sequence Read Archive (SRA) database under bioproject number PRJNA809514, and were set to be public on Apr 3. They can now be searched out in the NCBI SRA database.
